# Assessment of Suicide Risk in Patients with Depressive Episodes Due to Affective Disorders and Borderline Personality Disorder: A Pilot Comparative Study

**DOI:** 10.3390/brainsci14050463

**Published:** 2024-05-06

**Authors:** Elena Rudolfovna Isaeva, Daria Maksimovna Ryzhova, Anna Vladimirovna Stepanova, Ivo Nestorov Mitrev

**Affiliations:** 1 Department of General and Clinical Psychology, Academician I.P. Pavlov First St. Petersburg State Medical University, 197022 Saint Petersburg, Russia; daria.rmaks@yandex.ru; 2 10th Department—Center for Treatment of Depression and Anxiety, V. M. Bekhterev Psychiatry and Neurology National Medical Research Center, 192019 Saint Petersburg, Russia; stepany_81@mail.ru; 3 Department of Psychiatry and Medical Psychology, Medical University of Plovdiv, 4000 Plovdiv, Bulgaria; ivo.mitrev@mu-plovdiv.bg

**Keywords:** psychology of suicide, suicide risk, depression, personality disorders, attitude towards death

## Abstract

This study assessed suicidal risk in patients suffering from non-psychotic depressive disorders within various clinical and nosological forms (F31–F34 mood disorders and F60.31—emotionally unstable personality disorder). Clinical and psychological features were presented, as well as predictors of suicidal risk in patients of these groups. We performed a comparative analysis of the anxiety and depression level, the level of mental pain, fear of death and the severity of anti-suicidal motives in patients with affective disorders and borderline personality disorder (BPD). Based on the results, 100% of patients in these clinical nosological groups were found to have a high level of suicidal risk. Patients with affective disorders have weak anti-suicidal motives and are not fully aware of the consequences of their own death. Patients with BPD have a higher suicidal risk than patients with affective disorders; they are characterized by less pronounced social orientation, demonstrativeness, self-centeredness, less pronounced levels of anxiety and fear of death.

## 1. Introduction

Mental disorders, which can be called the main factors of suicide risk, are detected in more than 90% of suicide victims [1,2,3], and as a rule (approximately 80% of cases), do not have a timely diagnosis [4]. Using a cross-sectional design, Stoychev K et al. [5] accessed the medical records of all psychiatric patients who died by suicide over a 10-year period (2009–2018) in one major administrative region of Bulgaria. Here, 77 of 281 suicides (28%) had psychiatric records. The most common diagnoses were mood disorders (44%), followed by schizophrenia (27%), anxiety disorders (10%), substance use disorders (9%) and organic conditions (8%). Male gender, single/divorced marital status, early illness onset, co-occurring substance misuse and lower educational attainment (for patients aged below 70) were significantly associated with earlier age of suicide, whereas past suicide attempts and hospitalizations to mental clinics, co-morbid somatic conditions and unemployment showed an insignificant association. A substantial proportion of patients (60%) had contacted psychiatric service in the year preceding suicide, with nearly half of these encounters being within 30 days of the accident [5].

According to US suicidologists, mental disorders are diagnosed in 30% of people whose death is due to an act of suicide and in 40% of people who have suffered from incomplete suicide attempts. The risk of suicidal behavior in patients with affective disorders is maximum and exceeds the risk of suicide in patients with schizophrenia [6,7]. Numerous studies show that high suicidal risk is one of the main concomitants of depressive states [8,9]. Affective disorders account for at least 60% of suicides [10]. The most severe manifestations of auto-aggressive actions occur in psychopathological syndromes, in the structure of which depressive symptoms are the leading ones. According to British psychologists and psychiatrists, 41% of young people diagnosed with a depressive state of varying severity attempted suicide or performed self-destructive actions [11]. According to Russian studies [12,13], for example, in patients aged 17 to 25 years old suffering from depressive disorders of a non-psychotic level within various clinical nosological forms, an “auto-aggressive pattern” was identified, which allowed us to judge the possible algorithms of the behavior of the examined individuals. The suicidal intentions enactment associated with depression background depends to a greater extent not on the depth of the reaction but on the objective and subjective significance of mental trauma and the intensity of the experiences associated with it.

However, according to M.M. Linehan [14], in some cases, self-destructive actions should be considered in a parasuicidal context, such as “manipulative suicide attempts”, implying primarily the demonstrative orientation of self-destructive acts [15]. Such self-aggressive behavior is most often found in borderline personality disorder (BPD). According to M.C. Zanarini et al., from 46% to 92% of patients with BPD make suicide attempts, in which 10% of cases end in death [16]. According to M.M. Linehan, individuals suffering from BPD are characterized by emotional, behavioral and periodic cognitive dysregulation. High reactivity of emotions with episodic depression, anxiety, irritability, tense relationships with close associates and impulsiveness in decision making contribute to the formation of suicidal tendencies in people with borderline personality disorders [14,16].

According to a number of Russian researchers, from 20 to 40% of suicide attempts occur among psychopathic individuals. Borderline personality disorder (BPD) is a severe personality disorder that most often manifests in adolescence and young adulthood, characterized by decreased control over anger, intense and frequent mood swings, pronounced self-centeredness, risky behavior that threatens life and is the most suicidal among all personality disorders. Persons with emotionally unstable personality disorder of the borderline type have qualities such as impulsiveness, explosiveness and inability to control their own emotional outbursts. It is noted that in 87% of cases, suicide attempts in people with BPD were not planned in advance and occurred suddenly and impulsively as a response to a traumatic situation. In addition, the majority of suicidal people with borderline disorder note that suicidal experiences (thoughts, plans) accompanied them for a long time and, therefore, were perceived as the only option to solve problems [17,18].

Indeed, suicidal behavior in the overwhelming majority of cases is caused by the pronounced stressful nature of vital problems and the frustration of leading life needs [19]. According to V.F. Wojciech [20], about 75% of the younger generation die of suicide impulsively, and only 10% of them have true intentions to die. Often, the suffering subject does not strive for death as a goal but views it as a means of escaping life’s problems. It is important to take into account the following factors: living alone, divorce, the presence of severe, incurable somatic and mental illnesses, death of a loved one, admission to or discharge from a mental hospital, guilt, hopelessness, loneliness, lack of purpose or meaninglessness of life and substance abuse [21].

Authors [22] identify four groups of factors associated with suicide risk: personality and individual differences, cognitive factors, social factors and negative life events. Each of these factors can, independently or in combination, influence the probability of suicide risk occurrence.

The first group of factors includes personal traits such as impulsivity, perfectionism, neuroticism, extroversion, optimism, resilience and hopelessness.

In the group of cognitive factors, special attention should be given to fearlessness about injury and death and pain insensitivity, as suicide attempters demonstrate higher fearlessness about injury and death in comparison to control group participants.

It is important to highlight problem-solving and coping abilities, as research consistently shows a connection between suicidal behavior and deficits in interpersonal problem-solving and coping skills.

Rational pharmacotherapy combined with psychotherapy in affective (depressive, mixed and co-morbid) disorders is a powerful anti-suicidal factor, while preliminary identification of patients at high risk for suicide is mandatory, which currently seems problematic [23,24].

A number of studies have convincingly shown psychalgia (mental pain) to be one of the main predictors of suicidal actions [25,26]. It has been established that in individuals with a history of suicide attempts, the intensity of mental pain is significantly higher than in individuals who are not prone to suicide. The “Mental Pain Scale” is recognized as the most acceptable psychological construct for the general assessment of mental pain that has reached significant awareness [27] and can be used to assess suicidal risk [28].

The Reasons for Living Inventory method allows us to explore factors contributing to suicidal action prevention [14,29] and people with borderline disorders and suicide attempts history [14,30,31]. It should be noted that fear of death is a protective factor against suicidal acts. However, the fear of death is found to be significantly reduced in acute presuicide [32], which corresponds to T. Joyner’s theory of acquired fearlessness of death and the theory of destruction of the buffer of cultural anxiety [33]. Therefore, when assessing suicide risk, it is important to take into account both increased and decreased fears of death since they indicate mental distress and can lead to negative behavioral reactions. The “Fear of Personal Death” scale [32,34], which assesses rejection of this topic, also allows us to assess suicidal risk factors.

In Russian suicidology, there is a long overdue need for compact methods that cover these phenomena and promptly identify the risk groups [18,32]. The “Mental Pain Scale”, “Fear of Personal Death” and “Anti-Suicidal Motives Questionnaire”, in our opinion, are quite suitable for assessing the severity of suicidal risk.

The study objective was to assess the relevance of suicidal tendencies in patients suffering from non-psychotic depressive disorders within the framework of various clinical and nosological forms (mood disorders and emotionally unstable personality disorder) who were treated in a mental hospital.

## 2. Materials and Methods

The study was indicative in nature and was a pilot stage of a larger scientific study. A preliminary examination was made of 44 patients with F31–F34 and F60.31 who were being treated for a depressive episode in a mental hospital. As a result of a clinical interview and a biographical questionnaire, the subjects (24 people) with a history of suicide attempts (incomplete suicide) were excluded from the sampling. As a result, after selection, 20 subjects remained included in a pilot study aimed at identifying psychological markers of suicide risk in patients with emotional disorders of various origins. It should be noted that the general population of this type of patient sample does not have a large size, and it is a rather difficult task. In addition, many experimental designs do not involve large samplings (e.g., Robert Fisher, V.N. Druzhinin).

The sampling of the pilot study consisted of 20 subjects enrolled aged 19 to 46 years old. The average age of the participants was 25.6 ± 5.1 years. The criterion for inclusion in the sampling to conduct a psychological study was the following disorders coded according to ICD-10, established as a result of a clinical conversation by a psychiatrist: mood disorders [affective disorders] (F31–F34), emotionally unstable personality disorder of borderline type—PRL (F60.31). The diagnosis was made by the attending psychiatrist according to the diagnostic criteria of ICD-10 and DSM-5, and HADS questionnaires were used. The criterion for exclusion from the study was the patient’s history of suicide attempts.

The examination took place on the 30th day of the patients’ stay in a mental hospital in case the subjects gave voluntary informed consent.

To achieve the purpose of the study, the sampling was divided into two subgroups depending on the following existing diagnosis.

**Group No. 1**—patients with affective disorders (12 subjects), diagnosed as:

- BAR (F31.6)—3 subjects;
- Cyclothymia (F34.0)—2 subjects;
- Recurrent depression (F33.10)—3 subjects;
- Depressive episode (F32)—4 subjects.

**Group No. 2**—patients with emotionally unstable borderline personality disorder—F60.31 (8 subjects).

**The Objectives of the Study:**

1. To compare the degree of manifestation of depressive experiences of patients with affective disorders and patients with BPD.

2. To assess the relevance of suicidal thoughts and the severity of suicidal risk in the two compared groups of patients.

3. To substantiate the need to use these psychological techniques in the study of suicidal aspects of the personality of patients with affective disorders and borderline personality disorder, as well as to determine psychological markers of suicide risk.

### 2.1. Methods of Empirical Research

In accordance with the stated objective, the following psychodiagnostic methods, questionnaires and specialized clinical scales were used in the study.

I. To assess the severity of suicide risk, the “Mental Pain” Scale was used—an adapted Russian version [32] “The Psychache Scale (TPS)” [35] and the Columbia Suicidal Severity Scale (C-SSRS) [36].

II. To assess factors that prevent suicidal behavior, we used the Anti-Suicidal Motives Questionnaire—an adapted Russian version [37] of the “Reasons for Living Inventory” methodology [14] and the “Fear of Death” Scale—an adapted Russian-language version [12,38] of the “Fear of Personal Death Scale (FPDS)” methodology [34].

III. To study the clinical and psychological characteristics of the subjects, the Hospital Anxiety and Depression Scale (HADS), as well as a biographical questionnaire, were used.

### 2.2. Data Analysis Methods

Processing of quantitative data was carried out using Microsoft Excel 2016, as well as the IBM SPSS Statistics 23 statistical data processing software package.

Taking into account the small sample size of the pilot stage of the study, mathematical and statistical processing was carried out using non-parametric methods, which are used for small samples of subjects.

For the mathematical processing of data, the following methods were used: comparative analysis using the Mann–Whitney test to assess differences between groups, correlation analysis using Spearman’s rank correlation coefficient, as well as methods of descriptive statistics and analysis of average values.

## 3. Results and Discussion

### 3.1. Results Using the Hospital Anxiety and Depression Scale (HADS)

The Hospital Anxiety and Depression Scale (HADS) was used to assess the severity of emotional disturbances. The results revealed that 58.8% of the sample patients had a clinically significant level of anxiety, and 64.7% had a clinically significant level of depression.

**Figure 1:**
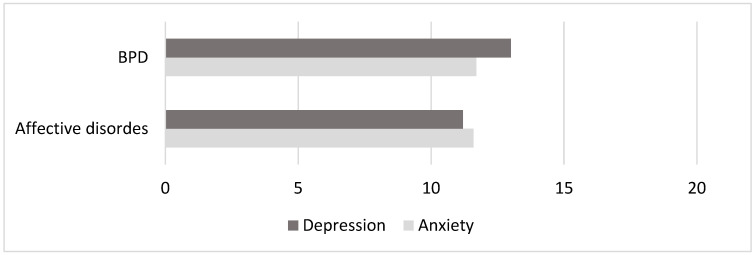
Anxiety and depression rates in two groups of patients. BPD, borderline personality disorder.

A comparative analysis of the average values of the level of anxiety and depression showed that anxiety in both affective disorders (m = 11.7 ± 2.7) and emotionally unstable personality disorder of the borderline type (m = 11.6 ± 3.3) was nearly within the same range, but the level of depression was slightly higher in BPD (m = 13 ± 4.3) (Figure 1). This result can be explained not by the severity of the disorder itself but by the higher emotional sensitivity of the patients with BPD, demonstrating traits in the personality structure, preoccupation with their experiences and a tendency to exaggerate them.

Despite the visible difference, the comparative analysis showed that this result is not statistically significant for this sampling (with *p* > 0.05).

### 3.2. Results Using the Columbia-Suicide Severity Rating Scale (C-SSRS)

To assess suicidal intentions, as well as the intensity, duration and frequency of suicidal thoughts, the Columbia Suicide Severity Rating Scale was used.

Analyzing the responses, 100% of the patients from the entire sample treated in a mental hospital due to a depressive episode were revealed to still have thoughts about the desire to die/stop living or about the desire to fall asleep and not to wake up.

For 23.5%, the main reason was an emotional state, which was expressed in “a feeling of unbearable reality”, powerlessness, fatigue, inner emptiness, “terrible hopelessness” and despair.

For 17.6%, they felt the desire not to wake up every day: “I get upset when I wake up”, “every night I fall asleep hoping not to wake up”.

Additionally, 11.8% of respondents had a desire for a “quiet death”, “to die without consequences”.

The answers obtained as a result of analyzing the emotional state of patients confirm the need to pay special attention to the degree of emotional distress: “What reasons did you have for thinking about the desire to die or suicide? Did you want to die to stop the pain or to stop experiencing what you were experiencing, or was your goal to get attention, revenge, or to get a response from other people? Or both?” Meanwhile, 100% of the subjects answered that they wanted to die of suicide mostly/exclusively in order to end their mental pain (score of 4 and 5 points).

**Figure 2:**
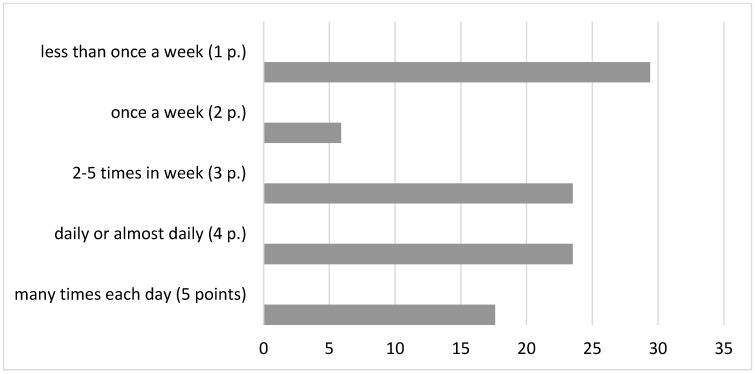
The incidence of suicidal ideas (%) in the general sampling; p—points.

Based on the results of the suicidal ideas intensity analysis, 35.3% of the subjects were revealed to have suicidal thoughts many times every day or almost every day (m = 4.3 ± 0.5), 29.4% of respondents noted such thoughts up to 2–5 times per week (m = 3 ± 0.1), while the remaining patients thought about suicide once per week or less (Figure 2).

**Figure 3:**
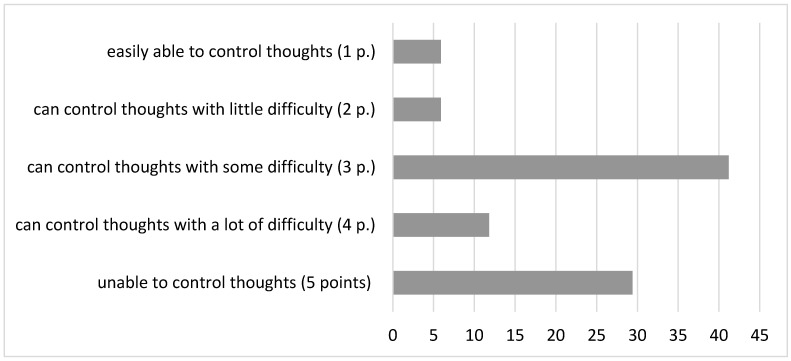
Control of suicidal thoughts (%) in the general sampling; p—points.

Almost half of respondents (47%) note that the duration of these thoughts is 1–8 h (long/most of the day), while 41% of patients are unable to control these thoughts or do it with great difficulty, the rest can exercise control with some minor difficulties (Figure 3).

Mann–Whitney comparative analysis did not reveal any statistical significance in the experience variations of suicidal thoughts and seriousness of intentions (*p* > 0.05) between the compared subgroups of patients: patients with affective disorders (m = 4.1 ± 1.4) and patients with emotionally unstable borderline personality disorder (m = 3 ± 1.6).

Thus, the results of the responses to the “Columbia Suicide Severity Rating Scale” analysis showed that most of the questions surveyed gave a score of 3 points or higher. Based on this, we can conclude that the majority of the examined patients have a serious (3–4 points) and high (5 points) risk of suicide. It is noteworthy that despite the fact that they underwent psychotherapeutic treatment for 1 month and received psychopharmacotherapy, a high and serious risk of suicide remained. The duration and severity of suicidal thoughts in patients during treatment are important to consider for psychiatrists and medical psychologists when choosing strategies for the pharmacological and psychotherapeutic treatment of these nosological groups of patients.

### 3.3. Results on the “Mental Pain” Scale

To assess the sensitivity threshold of mental pain, as well as determine its severity, the “Mental Pain” scale was used. A study of the level of mental pain in the compared subgroups of patients showed that both groups of patients have high scores on this scale (patients with affective disorders, m = 43.5 ± 10; patients with BPD, m = 41.6 ± 6.5, and for the maximum, 65), which reflects a fairly high level of experience.

**Figure 4:**
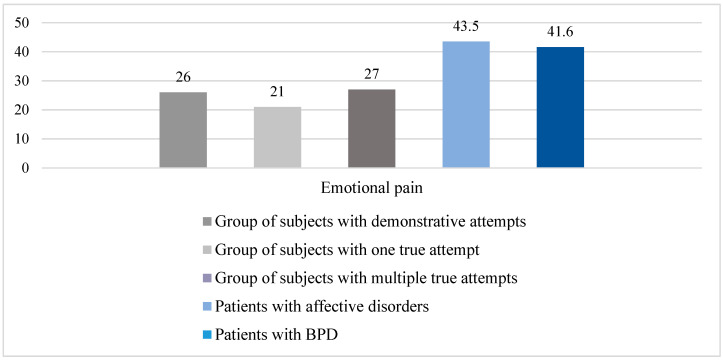
Average values on the Mental Pain scale in comparison with the data from T.V. Zhuravleva (2022) [7].

We compared our findings with the results of the study performed by a Russian psychologist from the Scientific Center for Mental Health (Moscow) T.V. Zhuravleva. “Psychological factors of suicidal behavior in young people with incomplete suicide attempts” (2022) [7]. The comparison results are presented in Figure 4.

It is noteworthy that the data obtained in our study on the level of mental pain in patients with a depressive episode in two nosological subgroups (F30–F34 and F60.31) turned out to be higher than in people with suicide attempts presented in the dissertation research of T.V. Zhuravleva. This may be due to the fact that those who attempted suicide found expression of their mental pain in the suicide attempt, and the patients in our study who did not have a history of suicide attempts while in a depressed state only thought about it and internally experienced the most intense mental pain. This contrasts with data from other authors who have argued that individuals with a history of suicide attempts have significantly higher levels of emotional pain than individuals who are not suicidal [28].

### 3.4. Results of the Anti-Suicidal Motives Questionnaire

**Figure 5:**
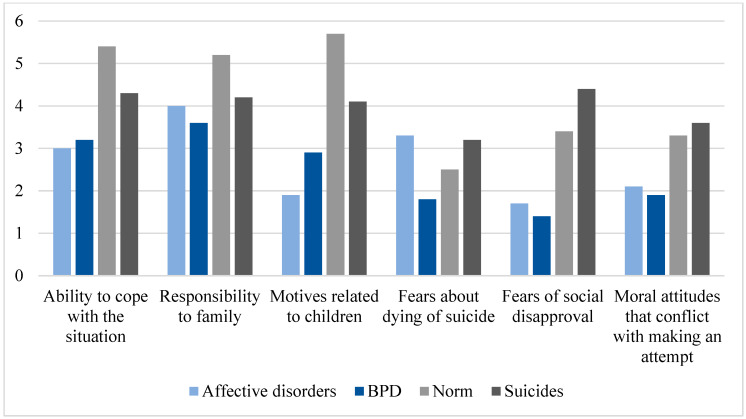
Distribution of average values of anti-suicidal motives in a sampling of patients with F30–F34 and F60.31, compared with suicides and the norm [37].

To assess the reasons preventing suicide, the Anti-Suicidal Motives Questionnaire was used. The results of the severity of anti-suicidal motives distribution on the scales showed the entire study sample to have a low level of severity of anti-suicidal motives; moreover, the average values of anti-suicidal motives of the subjects in our study are lower than the values of the suiciders samplings presented by the authors of the methodology (Figure 5).

However, the most significant anti-suicidal motive can be identified, both for patients with affective disorders and with emotionally unstable borderline personality disorder; this is “Responsibility to the family”.

The weakest anti-suicidal motive in both subgroups of patients was “Fear of social disapproval”, although increased rates for this motive are typical for individuals who have already made a suicide attempt. This can be associated both with the personal characteristics of the respondents and with the fact that the patients in our sample have not yet encountered condemnation or disapproval from others regarding suicidal behavior.

A significant difference was found in the motive “Fears about dying of suicide”: the average value in the subgroup of affective disorders is higher than in the subgroup of BPD (*p* < 0.05) but corresponds to the indicator of the suicides sampling. This difference is confirmed by the Mann–Whitney comparative analysis and is statistically significant.

Thus, both subgroups of patients have weakly expressed anti-suicidal motives; moreover, their values are lower than those of the suicide victims sampled by the authors of the Anti-Suicidal Motives Questionnaire. This observation echoes the results of other authors who noted that people in acute post-suicide fear both death and the act of suicide more than people without suicidal tendencies [7,12,38].

Also, there are statistically significant differences between the compared subgroups in the meaning of the motive “Fears about dying of suicide”: in patients with affective disorders, it is higher than in patients with BPD. This suggests that patients with BPD are less afraid of the attributes of suicide (blood, pain, violence) compared to patients with affective disorders.

### 3.5. Results of the Questionnaire “Fear of Personal Death”

**Figure 6:**
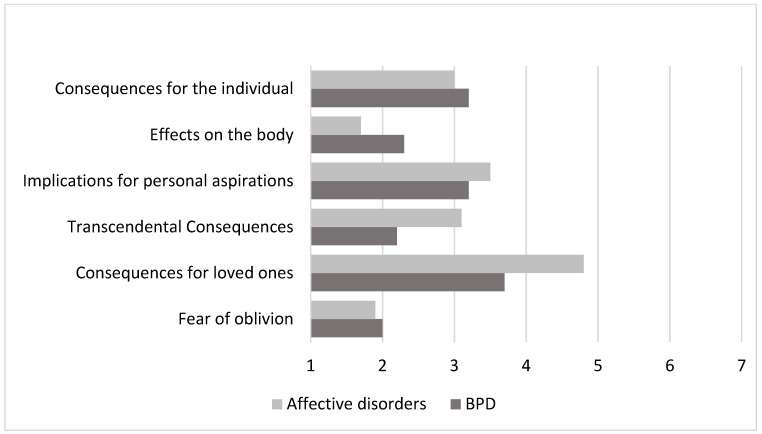
Average scale scores using the “Fear of Personal Death” method.

To assess patients’ awareness of the significance of the death consequences when intending to die of suicide, the “Fear of Personal Death” questionnaire was used. The significance of the consequences was assessed by the average indicators on a scale from 1 to 7, with an average of 4, but if the scale value is less than or equal to the average, then it is not significant. The results obtained are presented in Figure 6.

The results of the “Fear of Personal Death” questionnaire showed that both patients with affective disorders and patients with emotionally unstable borderline personality disorder do not fully understand the significance of the consequences of their own death. It means that in both subgroups, patients are not aware of the circumstances related to death and do not fully understand the consequences of their actions related to suicidal intentions. No statistically significant differences were found between the patient subgroups (*p* > 0.05).

### 3.6. Correlation Analysis

Subsequently, we conducted a Spearman correlation analysis in order to identify the relationship between the prosuicidal and anti-suicidal characteristics.

Thus, a relationship was found between anti-suicidal scales (Questionnaire of Anti-Suicidal Motives and Questionnaire “Fear of Personal Death”) and pro-suicidal scales (“Mental Pain” scale, “Columbia Suicide Severity Rating Scale”) at a significance coefficient level of 0.44, with *p* = 0.05 (Table 1).

Table 1 shows that the stronger the significance of anti-suicidal motives, the greater the patients’ fear of the consequences of their own death, such as consequences for the individual, loved ones, personal aspirations, as well as transcendental consequences and fear of oblivion. The strongest relationship is found with moral attitudes, which may serve as a deterrent that should be addressed when dealing with suicidal behavior. The Responsibility to Family and Fear of Social Disapproval scales are negatively correlated with the intention to carry out a suicide plan. This connection shows the high importance of social factors. That is, responsibility to the family and fear of social reaction reduce the patients’ intention to carry out their suicide plan.

## 4. Discussion

The analysis of the anxiety and depression level severity showed that anxiety in both affective disorders and emotionally unstable borderline disorder is the same. However, the level of depression in patients with BPD is slightly higher. We associate the obtained result with the personal characteristics of a subgroup of patients with BPD, such as higher emotional sensitivity, demonstrativeness, affective preoccupation with their experiences and a tendency to exaggerate them.

Using the Columbia Suicide Severity Rating Scale, the intensity, duration, frequency and controllability of suicidal ideation, as well as the severity of suicide intent, were examined in the study subgroups of patients. Based on the results obtained, the majority of the examined patients appear to have a serious (3–4 points) and high (5 points) risk of suicide. It should be noted that at the time of the examination, the patients were receiving psychiatric treatment, pharmacotherapy and psychotherapy, but despite this, 1 month after the treatment started, a high and serious risk of suicide still remained. This should be taken into account in the process of treatment and psychotherapeutic work with these groups of patients.

The study of the mental pain level in the compared subgroups showed that both subgroups of patients had high scores on the Mental Pain scale (43 out of 65), which reflects a fairly high intensity of the mental pain experience. There were no significant differences between subgroups in our study. However, when compared with data from other researchers [7], indicators of the mental pain level in the subgroups of patients with F30–F34 and F60.31, who never made suicide attempts turned out to be higher than in persons with incomplete suicide attempts. We explain this by the fact that suicidal people expressed their mental pain through suicide, and the patients in our study who were admitted to hospital treatment due to a depressive episode only thought about it and internally experienced the pain. This may indicate a high suicidal risk that persists despite treatment since all respondents think of suicide as an opportunity to stop the pain.

To study predictors preventing suicide, we studied patients’ responses using the Anti-Suicidal Motives Questionnaire and the Fear of Personal Death Scale. Considering the findings obtained, it was found that both subgroups of patients in our study have weakly expressed anti-suicidal motives; moreover, their values are lower than those of the suicide victims sampling the Questionnaire of Anti-Suicidal Motives authors [37]. However, it is possible to identify the most significant anti-suicidal motive for both subgroups of our study: it is “Responsibility to the family”. At the same time, statistically significant differences were observed in the importance of the motive “Fears of committing suicide”: in patients with BPD, it is lower than in patients with affective disorders. This suggests that patients with BPD are less afraid of the attributes of suicide (blood, pain, violence) compared to patients with affective disorders.

The results of the “Fear of Personal Death” questionnaire showed both patients with affective disorders and patients with emotionally unstable borderline personality disorder were not fully aware of the significance of the consequences of their own death.

The correlation analysis carried out in order to identify the relationship between pro-suicidal and anti-suicidal characteristics led to the conclusion that it is very important to pay attention to the ability to cope with the life difficulties of patients, as well as to the formation of social and moral attitudes since these characteristics can be protective factors for suicidal risk, they are the ones that reduce this risk.

## 5. Conclusions

1. In our study, 100% of patients suffering from non-psychotic depressive disorders within two clinical nosological forms (mood disorders and emotionally unstable personality disorder) had a high level of suicidal risk, despite the fact that at the time of the examination, they were being treated in a mental hospital and receiving pharmacotherapy and psychotherapy. This indicates the need for increased attention from specialists regarding the suicidal behavior of patients.

2. Patients with affective disorders are characterized by a tendency towards a weak expression of anti-suicidal motives: when considering suicide, they do not fully realize the consequences of their own death. Patients with emotionally unstable borderline personality disorder are less afraid of death and the process of attempting suicide than patients with affective disorders. 

3. Preliminary psychological predictors of suicidal risk in patients of both subgroups were identified: a high level of mental pain and a low level of fear of one’s own death. The following characteristics are likely to be protective factors that reduce suicidal risk: the severity of anti-suicidal motives, as well as the formation of social and moral attitudes.

4. Based on the total findings obtained in the pilot study, it can be stated with a sufficient degree of confidence that higher suicidal risk was observed in the subgroup of patients with emotionally unstable borderline personality disorder compared to patients with affective disorders. This may be due to the fact that patients with emotionally unstable personality disorder of the borderline type are distinguished by a less pronounced social orientation, demonstrativeness, self-centeredness, affective preoccupation with their experiences, a less pronounced level of anxiety and fear of death. This indicates a higher risk of suicide in this subgroup compared with patients with mood disorders.

## 6. Summary

Our data allowed us to study some psychological aspects of depressive experiences of patients with affective disorders and patients with borderline personality disorder. An attempt was made to assess the severity of suicidal tendencies in these groups and to identify predictors of suicidal behavior that specialists need to pay attention to both when assessing suicidal risk factors and when selecting psychopharmacological and psychotherapeutic treatment for patients with depressive disorders of various etiologies.

Insufficient psychodiagnosis and untimely detection of suicidal tendencies in patients suffering from depressive disorders of various origins can lead to disastrous consequences.

## 7. Limitations of the Study

Due to the fact that the article presents the results of the pilot stage of the study, in which the sampling was small, the results obtained are preliminary and cannot yet be extrapolated to the entire cohort of patients at risk of suicide. However, the identified trends provide grounds for a more thorough and comprehensive study of anti-suicidal and pro-suicidal motives as significant factors determining the level of suicide risk. Also, the findings obtained in our pilot study concern only psychiatric patients treated in hospital for a depressive episode (F31–F34 and F60.31) who did not have a history of suicide attempts. In this regard, it seems difficult to extrapolate the results obtained to samples of patients from other nosological groups.

## Figures and Tables

**Table 1:** Correlation among the Anti-Suicidal Motives Questionnaire, Fear of Personal Death, Mental Pain and the Columbia Suicide Severity Rating Scale, at *p* = 0.05. The significant correlational relationships are highlighted in red.

Scales of the Anti-Suicidal Motives Questionnaire	Columbia Suicide Severity Rating Scale						Mental Pain Scale
	Intention to Implement the SU Plan	SU Rate	Duration of SU	SU Control	Presence of Limiting Factors	SU Bases	
Ability to cope with situations	**−0.52**	**−0.56**	0.19	−0.43	**−0.48**	−0.37	**−0.56**
Responsibility to family	−0.23	−0.34	−0.21	0.05	0.03	−0.15	−0.13
Motives related to children	**−0.45**	−0.38	0.25	−0.07	−0.16	−0.04	−0.29
Fears about dying of suicide	−0.29	0.08	−0.06	−0.35	−0.29	−0.24	−0.09
Fears of social disapproval	**−0.49**	−0.34	0.02	−0.44	−0.2	−0.4	−0.42
Moral attitudes that conflict with making an attempt	−0.36	−0.38	−0.23	−0.28	**−0.45**	−0.34	−0.17
Mental Pain Scale	**0.5**	**0.77**	0.38	**0.66**	0.27	**0.51**	-
**Fear of Personal Death Scales**	**Columbia Suicide Severity Rating Scale**						**Mental Pain Scale**
	**Intention to implement the SU plan**	**SU rate**	**Duration of SU**	**SU control**	**Presence of limiting factors**	**SU bases**	
Consequences for the individual	−0.39	−0.27	0.03	−0.23	0.06	−0.27	−0.22
Effects on the body	−0.33	**−0.5**	−0.29	−0.38	−0.05	**−0.6**	−0.4
Implications for personal aspirations	−0.35	−0.37	−0.01	−0.25	−0.3	−0.4	−0.4
Transcendental Consequences	**−0.49**	−0.15	−0.18	−0.18	0.003	−0.18	−0.17
Consequences for loved ones	**−0.5**	−0.26	−0.07	−0.14	−0.11	−0.14	−0.21
Fear of oblivion	−0.41	−0.39	−0.1	−0.2	0.17	**−0.5**	−0.29

SU: suicide.

## Data Availability

This manuscript fully complies with the editorial and ethical policies. New data obtained from the research published in this article.
